# Association Between Metabolic Score for Insulin Resistance and the Incidence of Gastric Cancer in South Korea: A Nationwide Retrospective Study

**DOI:** 10.3390/jcm15072507

**Published:** 2026-03-25

**Authors:** Chi Hyeon Choi, Minkook Son, Jong Yoon Lee, Myeongseok Koh, Sang Yi Moon, Yeo Wool Kang

**Affiliations:** 1 Department of Internal Medicine, College of Medicine, Dong-A University, Busan 49201, Republic of Korea; hyunt23@dau.ac.kr (C.H.C.); kohdolchu@dau.ac.kr (M.K.); sang4401@dau.ac.kr (S.Y.M.); ywkang8756@dau.ac.kr (Y.W.K.); 2 Department of Physiology, College of Medicine, Dong-A University, Busan 49201, Republic of Korea

**Keywords:** insulin resistance, metabolic syndrome, gastric cancer

## Abstract

**Background/Objectives**: Insulin resistance (IR) is increasingly recognized as a factor associated with metabolic syndrome and various cancers. The metabolic score for insulin resistance (METS-IR) has emerged as a reliable surrogate marker for assessing IR. This study evaluated the association between the METS-IR and the gastric cancer (GC) incidence using data from a nationwide South Korean cohort. **Methods**: Data were obtained from the National Health Insurance Service (NHIS) cohort. A total of 318,336 participants aged ≥40 years who underwent a nationwide health screening between 2009 and 2010 were included and followed until GC diagnosis, death, or 31 December 2019. The METS-IR was calculated and categorized into quartiles. Hazard ratios (HRs) for GC incidence were estimated using Cox proportional hazards models. The analyses were adjusted for confounders, including age, sex, socioeconomic status, lifestyle factors, and comorbidities. **Results**: Participants in the highest METS-IR quartile (Q4) exhibited a significantly higher crude incidence of GC (2.26 per 1000 person-years) than those in the lowest quartile (Q1: 1.97 per 1000 person-years). Adjusted HRs showed a modest but statistically significant increase in GC risk in Q4 (HR: 1.10; 95% confidence interval: 1.02–1.19; *p* = 0.01) compared to Q1. Kaplan–Meier analysis revealed that participants with higher METS-IR levels had significantly shorter GC-free survival times than those in the lower quartiles. Restricted cubic spline analysis revealed a nonlinear relationship between the METS-IR and GC risk, with higher METS-IR levels associated with an increased risk. **Conclusions**: An elevated METS-IR was associated with an increased GC risk, suggesting its potential utility in stratifying GC risk. The METS-IR may help identify high-risk individuals and support GC prevention.

## 1. Introduction

Gastric cancer (GC) is among the most commonly diagnosed malignancies worldwide, ranking fifth in incidence among all cancer types and serving as the third leading cause of cancer-related mortality [1]. In South Korea, GC ranks fourth in incidence and remains the third leading cause of cancer-related deaths, reflecting a significant disease burden in this region [2]. The development of GC is influenced not only by *Helicobacter pylori* infections but also by a complex interaction of environmental, host, and genetic factors. Additionally, several dietary and lifestyle habits have been linked to an increased risk of gastric carcinogenesis. These include smoking, high salt intake, excessive carbohydrate consumption, vitamin C deficiency, and a low carotenoid-rich vegetable intake [3,4,5]. Many of these factors contribute to metabolic abnormalities and provide a mechanistic link between lifestyle habits and GC. Metabolic syndrome (MetS) is characterized by metabolic abnormalities such as obesity, hypertension, and dyslipidemia. Insulin resistance (IR) plays a central role in both MetS and obesity. Growing evidence suggests that IR is associated with an increased risk of several cancers, including lung, prostate, colorectal, and breast cancer [6,7]. Although the relationship between IR and GC has not yet been clearly established, the gastric mucosa is highly sensitive to continuous regeneration and inflammatory stimuli. Therefore, IR-induced changes such as chronic inflammation, increased cell proliferation, and inhibition of apoptosis may contribute to the development of GC. The gold standard method for evaluating IR is the hyperinsulinemic-euglycemic clamp test [8,9]; however, owing to its technical complexity, invasive nature, and need for specialized equipment, it is not widely used in clinical practice [10]. Therefore, various surrogate markers have been developed to indirectly assess IR without directly measuring insulin levels. Among these, the Metabolic Score for insulin resistance (METS-IR) is a convenient and noninvasive index calculated using serum lipid profiles and body mass index (BMI), providing a rapid and practical method for estimating IR. Previous studies have examined the relationship between surrogate markers of IR and cancer risk and have shown that increased levels of these markers are associated with an increased risk of cancer [11,12]. However, no studies to date have investigated the relationship between METS-IR, a surrogate marker of IR, and the incidence of GC or analyzed subgroup differences based on sex and age.

## 2. Methods

### 2.1. Study Design and Population

This retrospective cohort study analyzed the claims data from the National Health Insurance Service (NHIS) National Health Screening Cohort (NHIS-HealS) in South Korea. The NHIS is a government insurance agency providing mandatory insurance to the South Korean population. The National Health Screening Program (NHSP) is provided biennially to insured individuals aged ≥40 years and includes physical examinations, blood tests, urine tests, and upper gastrointestinal endoscopy examinations. The collected data included test results, sociodemographic factors, and lifestyle behavior information obtained through questionnaires, all of which were systematically documented and stored.

The NHIS-HealS database integrates information regarding population statistics, prescription medications, medical procedures, treatment claims, and diagnoses based on the International Classification of Diseases 10th edition (ICD-10) codes. In this study, we identified 377,641 individuals aged ≥40 years who participated in the NHSP between 2009 and 2010 using the NHIS-HealS database.

Inclusion criteria:

- Individuals aged ≥40 years who participated in the NHSP between 2009 and 2010 (*n* = 377,641).

Exclusion criteria:

- Individuals with a prior diagnosis of GC (*n* = 5504).
- Individuals diagnosed with other during follow-up (*n* = 34,780).
- Individuals with incomplete data (*n* = 3972).
- Individuals with extreme METS-IR values (outside the 1st and 99th percentiles) (*n* = 15,049).

After applying these criteria, the final study cohort comprised 318,336 individuals.

**Figure 1:**
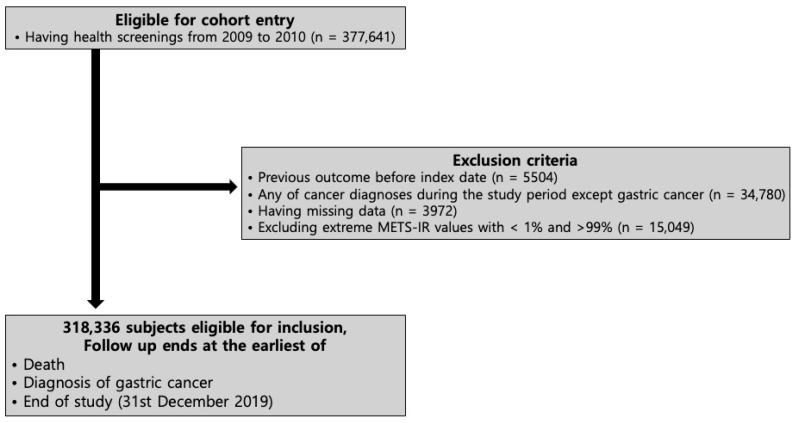
Flow diagram of participant selection in the study cohort. A total of 377,641 participants aged ≥40 years from the 2009–2010 NHSP were identified. After excluding individuals with prior GC, other cancers, incomplete data, or extreme METS-IR values, 318,336 participants were included and followed up until GC diagnosis, death, or 31 December 2019.

Participants were followed from the baseline health screening until the occurrence of GC, death, or 31 December 2019, whichever occurred first (Figure 1). The follow-up end date was determined based on the latest year for which complete and validated outcome data were available in the NHIS database at the time of analysis. Individuals who underwent health screening during 2009–2010 were selected as the baseline cohort to ensure a sufficiently long follow-up period for evaluating the incidence of GC, which typically requires long-term observation.

### 2.2. Definitions for Key Variables

The METS-IR was evaluated based on triglyceride (TG), fasting blood glucose (FBG), and high-density lipoprotein cholesterol (HDL-C) levels and BMIs.

The METS-IR was calculated using the following formula:

ln[(2 × FPG (mg/dL) + TG (mg/dL)) × BMI (kg/m^2^)]/ln[HDL-C (mg/dL)]

Participants were categorized into quartiles (Q1–Q4) based on METS-IR levels, with Q1 representing the lowest quartile and Q4 representing the highest. To identify individuals with newly diagnosed GC for data analysis, we utilized NHIS primary data, with a primary focus on insurance claims. Patients with GC were identified based on the ICD-10 diagnostic code C16 in the NHIS claims data.

### 2.3. Definitions of Covariates

The baseline covariates included demographic factors, lifestyle behaviors, and clinical variables. The Charlson Comorbidity Index (CCI) was determined based on existing comorbid conditions [13]. BMIs were calculated by dividing the body weight (kg) by the height squared (m^2^). According to the World Health Organization’s Asia-Pacific guidelines, obesity was defined as a BMI of ≥25 kg/m^2^. The laboratory parameters included hemoglobin levels and glomerular filtration rates (GFRs). A self-reported questionnaire was used to collect information on smoking status, alcohol intake, and regular physical activity. Income levels were divided into four quartiles, and residential areas were categorized as urban or rural. The criteria for hypertension, diabetes mellitus, and dyslipidemia are listed in Supplementary Table S1.

### 2.4. Statistics and Data Analysis

Descriptive statistics were used to summarize baseline characteristics across the METS-IR quartiles. Continuous variables were compared using analysis of variance, and categorical variables were compared using the chi-squared test; for ordered categorical variables, the linear-by-linear chi-square test was used to assess trends across categories. Kaplan–Meier curves were constructed to evaluate GC-free survival, and differences between quartiles were tested using the log-rank test. Cox proportional hazards models were used to calculate the hazard ratios (HRs) and 95% confidence intervals (CIs) for the association between METS-IR and GC incidence. The models were adjusted for age, sex, income level, residence, smoking, alcohol use, regular exercise, BMI, hemoglobin level, GFR, hypertension, diabetes mellitus, dyslipidemia, and CCI. Because death may preclude the occurrence of GC, competing risk analysis was additionally performed using the Fine–Gray subdistribution hazard model, treating death as a competing event, and subdistribution HRs with 95% CIs were estimated. Restricted cubic splines were used to examine potential nonlinear associations between the METS-IR and GC risk. All analyses were performed using SAS (version 9.4) and R (version 4.0.3), with a *p*-value < 0.05 considered statistically significant.

### 2.5. Ethics Statement

This study was approved by the Institutional Review Board of the Dong-A University College of Medicine and adhered to the principles of the Declaration of Helsinki. The requirement for informed consent was waived due to the retrospective nature of the study and the use of anonymized data.

## 3. Results

### 3.1. Baseline Characteristics

Table 1 summarizes the distributions of demographic, clinical, and lifestyle factors across the METS-IR quartiles. The number of participants in each quartile was 79,572 in Q1, 79,661 in Q2, 79,531 in Q3, and 79,572 in Q4. Higher METS-IR quartiles were associated with older age, male sex, and a higher prevalence of hypertension, diabetes mellitus, and dyslipidemia. BMI, blood pressure, FBG, TG, and low-density lipoprotein cholesterol (LDL-C) levels were significantly higher in Q4 than in Q1. Conversely, HDL-C levels and GFRs were lower in Q4 than in Q1. The smoking and alcohol consumption rates were higher in Q4, whereas regular exercise was less common.

### 3.2. Incidence of GC According to the METS-IR

**Figure 2:**
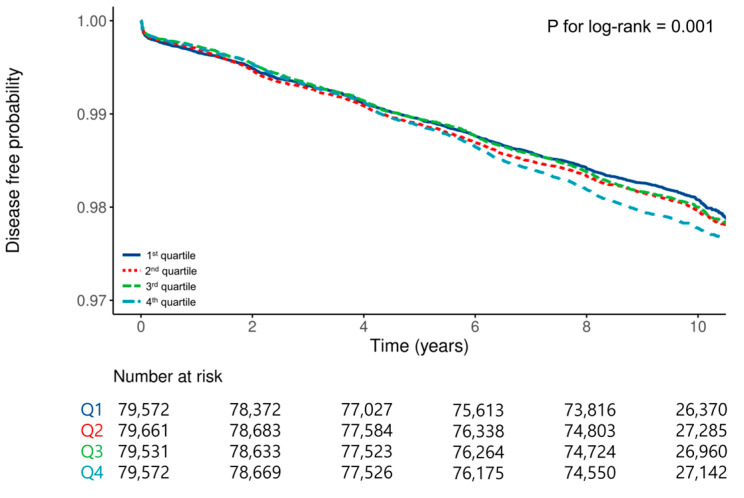
Kaplan–Meier curves for gastric cancer-free survival according to Metabolic Score for Insulin Resistance quartiles.

**Figure 3:**
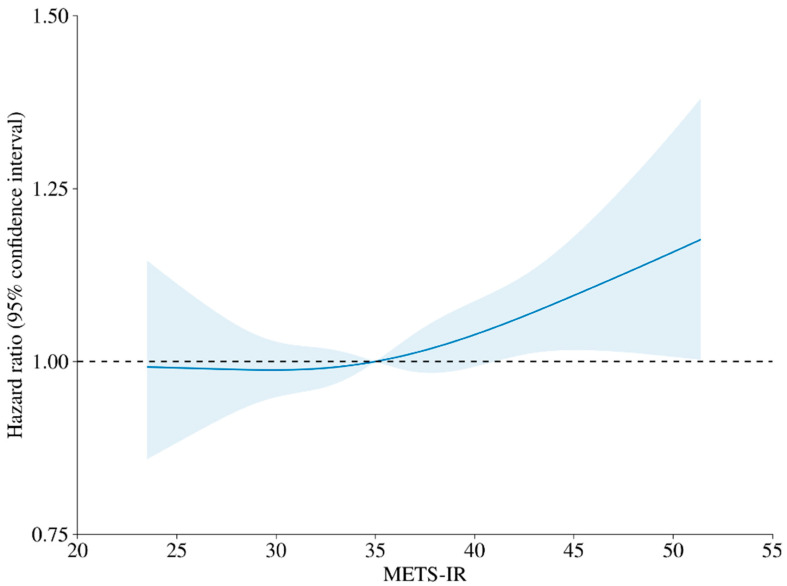
Restricted cubic spline curve for the association between Metabolic Score for Insulin Resistance and gastric cancer risk. The solid line represents the hazard ratio, and the shaded area indicates the 95% confidence interval. The dashed horizontal line indicates the reference value (HR = 1).

Table 2 presents the crude and adjusted HRs with 95% CIs for the relationship between METS-IR quartiles and GC risk. The crude incidence rates of GC increased across the METS-IR quartiles: 1.97 cases per 1000 person-years in Q1, 2.08 in Q2, 2.05 in Q3, and 2.26 in Q4. Cox proportional hazards analysis revealed that, after adjusting for confounders, participants in Q4 had a significantly higher risk of GC compared to Q1 (adjusted HR: 1.10; 95% CI: 1.02–1.19; *p* = 0.01). In the Fine–Gray competing risk regression model treating death as a competing event, participants in Q4 had a significantly higher risk of GC compared with those in Q1 after adjustment for confounders (adjusted HR: 1.13; 95% CI: 1.05–1.22; *p* = 0.002). Kaplan–Meier survival analysis demonstrated significantly shorter GC-free survival times in Q4 than in Q1 (log-rank *p* < 0.001) (Figure 2). Restricted cubic spline analysis revealed a nonlinear relationship between the METS-IR and GC risk, with a continuous increase in risk beyond a certain threshold. Specifically, when the METS-IR exceeded the mean value of 35, the HR increased to >1, indicating a higher risk of GC. Furthermore, the 95% CI surpassed 1 at a METS-IR value of 41.2, suggesting a significant association between elevated METS-IR levels and increased gastric cancer risk (Figure 3).

Kaplan–Meier survival analysis showed significantly shorter GC-free survival in participants in the highest METS-IR quartile compared with those in the lowest quartile (log-rank *p* < 0.001).

Restricted cubic spline analysis showed a nonlinear increase in GC risk with higher METS-IR levels, exceeding the reference (HR > 1) at values above 35.

### 3.3. Subgroup Analysis

**Figure 4:**
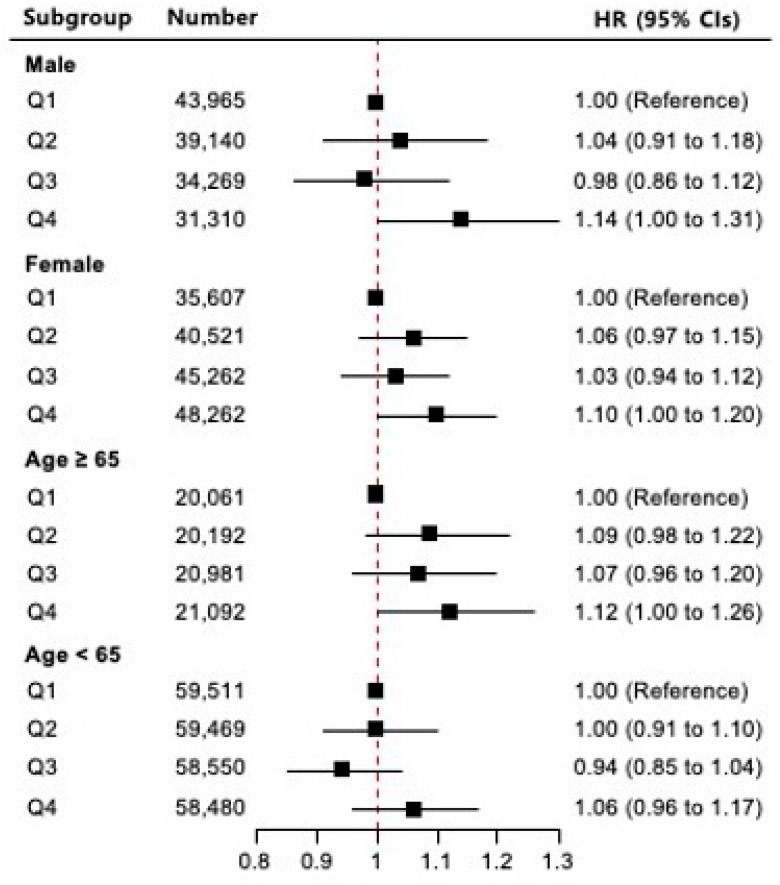
Hazard ratio (HR) and 95% confidence interval (CI) for incidence of gastric cancer according to metabolic score for insulin resistance quantiles in subgroup analyses stratified by sex and age. Squares represent HR, and horizontal lines indicate 95% CI. The dashed vertical line represents the reference value (HR = 1).

Figure 4 presents the HRs or GC incidence according to the METS-IR quantiles in the subgroup analyses stratified by sex and age. In the sex-stratified analysis, males in Q4 had a significantly increased risk of GC compared to Q1 (adjusted HR: 1.14; 95% CI: 1.00–1.31; *p* = 0.05). In contrast, among females, no statistically significant association was observed between the METS-IR and GC risk (adjusted HR for Q4 vs. Q1: 1.10; 95% CI: 1.00–1.20; *p* = 0.05). In the age-stratified analysis, individuals aged ≥65 years showed a borderline significant association between METS-IR and GC risk, with an adjusted HR of 1.12 (95% CI: 1.00–1.26; *p* = 0.05) in Q4 compared to Q1. However, in individuals aged <65 years, Q4 was not significantly associated with an increased risk of GC (adjusted HR: 1.06; 95% CI: 0.96–1.17; *p* = 0.25).

## 4. Discussion

This is the first large-scale nationwide population-based cohort study to evaluate the association between the METS-IR, a surrogate marker of IR, and GC incidence. Using various statistical methods, we confirmed that the METS-IR showed a significant positive association with the incidence of GC, supporting its role as a valid surrogate marker for IR. This result is consistent with that of a previous cohort study conducted in Korea, which demonstrated a similar association between the triglyceride-glucose (TyG) index and GC incidence [14]. 

Although studies investigating the association between IR surrogate markers and GC remain limited, IR has been recognized as a significant contributor to the development of several cancers, including colorectal and other obesity-related malignancies [11,15,16,17].

Several mechanisms have been proposed to explain how IR promotes carcinogenesis. Insulin promotes cellular proliferation, and this mitogenic effect is further enhanced under conditions of IR-associated hyperinsulinemia. Additionally, insulin exerts anti-apoptotic effects by suppressing programmed cell death in DNA-damaged cells, thereby facilitating carcinogenesis. These mechanisms have been demonstrated in breast cancer models, where the overexpression of insulin receptor substrate-1 has been observed. Adipose tissue-derived proteins, such as leptin, which are stimulated by insulin, have been shown to promote cell proliferation and anti-apoptotic activity. In contrast, adiponectin levels decrease in IR and obesity, and this protein is thought to counteract tumor-promoting processes [18,19,20]. Furthermore, previous studies have reported a positive association between surrogate markers of IR and *Helicobacter pylori* infection [21]. As *Helicobacter pylori* infection is a well-established risk factor for GC, this finding may support a mechanistic link between IR and gastric carcinogenesis.

Subgroup analyses according to sex and age revealed that the association between METS-IR and GC was more pronounced in males and in older adults. This finding is consistent with those of prior studies suggesting that aging is associated with progressive metabolic decline, which may contribute to increased IR and cancer risk [6,7,11,12]. Advanced age is typically accompanied by a reduction in basal metabolic rate, decreased muscle mass, lower insulin sensitivity, and hormonal changes, all of which may contribute to worsening metabolic dysfunction and increased IR, thereby promoting cancer development. A greater accumulation of visceral fat and lower levels of insulin sensitivity are more commonly observed in men than in women. Moreover, men are more likely to engage in unhealthy lifestyle behaviors, such as smoking, alcohol consumption, and poor dietary habits, all of which are known to contribute to chronic inflammation, oxidative stress, and metabolic disturbances that can facilitate carcinogenesis. Epidemiological evidence has consistently shown that men are more susceptible to metabolism-related cancers, including those of the gastrointestinal tract, and tend to have worse prognoses than women [22,23]. Therefore, the stronger association observed in male and older adult subgroups may reflect both biological susceptibility and lifestyle-related risk factors. These findings highlight the potential value of the METS-IR as a practical clinical tool for identifying high-risk individuals, especially older adults and men, who may benefit from targeted cancer prevention strategies and early intervention programs.

This study had several limitations. First, the NHIS database does not include information on *Helicobacter pylori* infections, which limits our ability to evaluate causal relationships between IR and GC. Future studies should incorporate serological and histological data to account for *Helicobacter pylori* infection and eradication status. Second, this study did not classify GC by histological subtype (e.g., intestinal vs. diffuse type). Because the risk factors and pathophysiology may differ between subtypes, future studies should be conducted at a single time point, making it difficult to evaluate long-term changes in metabolic status, medication use, or lifestyle factors over a 10-year follow-up period. Repeated measurements are required in future studies to better understand the effects of metabolic changes over time. Moreover, this study used claims data of Korean adults, which may limit its generalizability to other populations. Because participants in the NHSP may be more health-conscious than the general population, selection bias may have occurred. Important confounding factors such as a family history of cancer, dietary habits, physical activity, and alcohol consumption were not available in the dataset, leaving the possibility of residual confounding. In addition, GC was not classified according to anatomical subsite (cardia vs. non-cardia) due to limitations of the database. Given the distinct etiologies of these subtypes—where cardia cancer is more closely associated with obesity-related factors such as gastroesophageal reflux disease, and non-cardia cancer is primarily linked to chronic inflammation and *Helicobacter pylori* infection—this lack of stratification may have influenced the observed associations [24]. Despite these limitations, this study is significant, as it is the first to investigate the relationship between METS-IR and the incidence of GC in a large-scale population-based cohort. By leveraging comprehensive national health data and applying robust statistical methodologies, we were able to provide reliable evidence supporting the potential link between metabolic dysfunction and gastric carcinogenesis. The large sample size and long follow-up duration enhance the statistical power and generalizability of our findings, which underscores the potential clinical utility of METS-IR not only as a surrogate marker of IR but also as a novel risk stratification tool for identifying individuals at increased risk of developing GC. Future prospective studies and external validation across diverse populations are essential to confirm these findings and further explore the mechanisms underlying this association. Elevated METS-IRs were significantly associated with increased risk of GC, suggesting its role as both a mechanistic link and a potential clinical marker for identifying high-risk individuals.

## 5. Conclusions

Higher METS-IR was associated with an increased risk of GC in this nationwide cohort study. These findings suggest that insulin resistance may play a role in gastric carcinogenesis and highlight the potential value of METS-IR as a risk stratification marker.

## Figures and Tables

**Table 1:** Baseline characteristics of study population according to METS-IR.

All Subjects (*N* = 318,336)	METS-IR				*p*-Value
	1st Quartile, Q1 (*N* = 79,572)	2nd Quartile, Q2 (*N* = 79,661)	3rd Quartile, Q3 (*N* = 79,531)	4th Quartile, Q4 (*N* = 79,572)	
Demographics					
Age (years)	58.7 (9.1)	58.9 (8.6)	59.2 (8.5)	59.2 (8.4)	<0.001
Sex (%)					<0.001
Male	35,607 (44.7)	40,521 (50.9)	45,262 (56.9)	48,262 (60.7)	
Female	43,965 (55.3)	39,140 (49.1)	34,269 (43.1)	31,310 (39.3)	
Income level (%)					<0.001
1st quartile	11,876 (14.9)	11,193 (14.1)	10,866 (13.7)	10,752 (13.5)	
2nd quartile	17,889 (22.5)	16,629 (20.9)	15,594 (19.6)	15,564 (19.6)	
3rd quartile	22,921 (28.8)	23,537 (29.5)	23,560 (29.6)	24,314 (30.6)	
4th quartile	26,886 (33.8)	28,302 (35.5)	29,511 (37.1)	28,942 (36.4)	
Residence (%)					<0.001
Urban	52,075 (65.4)	51,597 (64.8)	51,127 (64.3)	49,987 (62.8)	
Rural	27,497 (34.6)	28,064 (35.2)	28,404 (35.7)	29,585 (37.2)	
Underlying disease					
Hypertension (%)	26,128 (32.8)	34,249 (43.0)	40,612 (51.1)	48,507 (61.0)	<0.001
Anti-hypertensive drugs (%)	20,950 (26.3)	28,140 (35.3)	34,008 (42.8)	41,576 (52.3)	<0.001
Diabetes (%)	4877 (6.1)	8378 (10.5)	12,447 (15.7)	18,728 (23.5)	<0.001
Anti-diabetic drugs (%)	3772 (4.7)	6520 (8.2)	9460 (11.9)	13,794 (17.3)	<0.001
Dyslipidemia (%)	19,285 (24.2)	26,487 (33.2)	33,673 (42.3)	46,494 (58.4)	<0.001
Charlson comorbidity index					<0.001
0	41,475 (52.1)	37,771 (47.4)	35,615 (44.8)	32,018 (40.2)	
1	21,560 (27.1)	22,187 (27.9)	21,970 (27.6)	21,616 (27.2)	
2	9588 (12.0)	10,706 (13.4)	11,218 (14.1)	12,285 (15.4)	
≥3	6949 (8.7)	8997 (11.3)	10,728 (13.5)	13,653 (17.2)	
Health screening					
Body mass index (kg/m^2^)	21.0 (1.5)	23.3 (1.4)	24.8 (1.5)	27.0 (2.1)	<0.001
Systolic blood pressure (mmHg)	121.4 (15.3)	124.6 (15.0)	126.8 (14.8)	128.9 (14.7)	<0.001
Diastolic blood pressure (mmHg)	75.3 (9.8)	77.0 (9.8)	78.4 (9.7)	79.7 (9.7)	<0.001
Fasting blood glucose (mg/dL)	94.1 (15.9)	97.9 (19.2)	101.9 (22.5)	107.9 (28.1)	<0.001
Total cholesterol (mg/dL)	199.2 (35.5)	201.0 (37.0)	201.3 (37.8)	200.2 (38.1)	<0.001
Triglyceride (mg/dL)	94.1 (41.5)	117.4 (52.8)	142.6 (65.0)	182.1 (79.8)	<0.001
HDL cholesterol (mg/dL)	63.5 (16.3)	55.8 (11.2)	50.8 (10.1)	45.1 (9.5)	<0.001
LDL cholesterol (mg/dL)	117.1 (35.3)	121.6 (35.5)	122.1 (37.6)	118.9 (37.7)	<0.001
Hemoglobin (g/dL)	13.4 (1.4)	13.7 (1.4)	14.0 (1.5)	14.2 (1.5)	<0.001
Glomerular filtration rate (mL/min/1.73 m^2^)	80.3 (29.0)	79.2 (31.3)	77.8 (30.5)	76.9 (32.8)	<0.001
Smoking (%)					<0.001
Non-smoker	56,304 (70.8)	53,658 (67.4)	50,543 (63.6)	48,010 (60.3)	
Ex-smoker	10,242 (12.9)	13,680 (17.2)	15,824 (19.9)	16,935 (21.3)	
Current smoker	13,026 (16.4)	12,323 (15.5)	13,164 (16.6)	14,627 (18.4)	
Alcohol drinking (%)	28,863 (36.3)	31,074 (39.0)	32,695 (41.1)	32,950 (41.4)	<0.001
Regular exercise (%)					<0.001
No	55,090 (69.2)	53,248 (66.8)	52,800 (66.4)	53,331 (67.0)	
1–2 times/week	14,348 (18.0)	15,331 (19.3)	15,653 (19.7)	15,758 (19.8)	
3–4 times/week	6115 (7.7)	6755 (8.5)	6927 (8.7)	6578 (8.3)	
≥5 times/week	4019 (5.1)	4327 (5.4)	4151 (5.2)	3905 (4.9)	
METS-IR	28.7 (1.9)	33.3 (1.0)	36.9 (1.1)	42.6 (2.9)	<0.001

**Table 2:** Hazard ratio and 95% confidence interval for incidence of gastric cancer according to metabolic score for insulin resistance.

Subjects (*N* = 318,336)	Events	Follow-Up Duration (Person-Years)	Incidence Rate (per 1000 Person-Years)	Hazard Ratio (95% Confidence Intervals)	
				Crude	Adjusted *
Q1 (*N* = 79,572)	1472	746,300	1.97	1 (reference)	1 (reference)
Q2 (*N* = 79,661)	1567	753,575	2.08	1.05 (0.98–1.13, *p* = 0.14)	1.05 (0.98–1.13, *p* = 0.18)
Q3 (*N* = 79,531)	1545	752,753	2.05	1.04 (0.97–1.12, *p* = 0.27)	1.01 (0.94–1.09, *p* = 0.76)
Q4 (*N* = 79,572)	1701	752,612	2.26	1.15 (1.07–1.23, *p* < 0.001)	1.10 (1.02–1.19, *p* = 0.01)

* The model was adjusted for age, sex, income level, residence, hypertension, diabetes, dyslipidemia, Charlson comorbidity index, hemoglobin level, glomerular filtration rate, smoking, alcohol drinking, and regular exercise status.

## Data Availability

The data used in this study are available from the National Health Insurance Service (NHIS) of Korea but restrictions apply to the availability of these data, which were used under license for the current study.

## Supplementary Materials

The following supporting information can be downloaded at: https://www.mdpi.com/article/10.3390/jcm15072507/s1, Supplementary Table S1. Definitions for clinical variables.

## Abbreviations

GC	Gastric Cancer
MetS	Metabolic Syndrome
IR	Insulin Resistance
METS-IR	Metabolic Score for Insulin Resistance
NHSP	National Health Screening Program
NHIS	National Health Insurance Service
NHIS-HealS	National Health Screening Cohort
TG	Triglyceride
HDL-C	High-Density Lipoprotein Cholesterol
TyG Index	Triglyceride-Glucose Index
LDL-C	Low-Density Lipoprotein Cholesterol
FBG	Fasting Blood Glucose
CCI	Charlson Comorbidity Index
BMI	Body Mass Index
GFR	Glomerular Filtration Rate
ICD	International Classification of Diseases
