# Supplementary material for: Association Between Metabolic Score for Insulin Resistance and the Incidence of Gastric Cancer in South Korea: A Nationwide Retrospective Study
Source: J Clin Med. 2026 Mar 25;15(7):2507. doi: 10.3390/jcm15072507 (PMC13073363; doi:10.3390/jcm15072507)
Supplement: Supplementary file 1 [file jcm-15-02507-s001.zip › jcm-4170758-supplementary.pdf]

Supplementary Table S1. Definitions for clinical variables.

Clinical variables	ICD-10 codes + Claim codes	Health screening
Hypertension	I10-I11 + Prescription of anti-hypertensive drugs ≥ 1	Systolic blood pressure ≥ 140 mmHg or<br>Diastolic blood pressure ≥ 90 mmHg
Diabetes	E10-E14 + Prescription of anti-diabetic drugs ≥ 1	Fasting blood glucose ≥ 126 mg/dL
Dyslipidemia	E78 + Prescription of lipid-lowering drugs ≥ 1	Total cholesterol 240 mg/dL or<br>LDL cholesterol 190 mg/dL or<br>Triglyceride 500 mg/dL or<br>HDL cholesterol < 40 mg/dL
Gastric cancer	C16	
